# Control of Biofilm Formation in Healthcare: Recent Advances Exploiting Quorum-Sensing Interference Strategies and Multidrug Efflux Pump Inhibitors

**DOI:** 10.3390/ma11091676

**Published:** 2018-09-10

**Authors:** Bindu Subhadra, Dong Ho Kim, Kyungho Woo, Surya Surendran, Chul Hee Choi

**Affiliations:** Department of Microbiology and Medical Science, Chungnam National University School of Medicine, Daejeon 35015, Korea; bindu.subhadra@gmail.com (B.S.); casiopea1208@naver.com (D.H.K.); khwoo1991@gmail.com (K.W.); tankz93@gmail.com (S.S.)

**Keywords:** biofilm formation, healthcare, biofilm inhibition, quorum sensing, multidrug efflux pumps

## Abstract

Biofilm formation in healthcare is an issue of considerable concern, as it results in increased morbidity and mortality, imposing a significant financial burden on the healthcare system. Biofilms are highly resistant to conventional antimicrobial therapies and lead to persistent infections. Hence, there is a high demand for novel strategies other than conventional antibiotic therapies to control biofilm-based infections. There are two approaches which have been employed so far to control biofilm formation in healthcare settings: one is the development of biofilm inhibitors based on the understanding of the molecular mechanism of biofilm formation, and the other is to modify the biomaterials which are used in medical devices to prevent biofilm formation. This review will focus on the recent advances in anti-biofilm approaches by interrupting the quorum-sensing cellular communication system and the multidrug efflux pumps which play an important role in biofilm formation. Research efforts directed towards these promising strategies could eventually lead to the development of better anti-biofilm therapies than the conventional treatments.

## 1. Introduction

Biofilms are surface-attached groups of microbial cells that are embedded in a self-produced extracellular matrix and are highly resistant to antimicrobial agents [1,2,3]. Biofilms can attach to all kinds of surfaces, including metals, plastics, plant and body tissue, medical devices and implant materials [4]. Biofilm formation on indwelling medical devices and implants such as heart valves, pacemakers, vascular grafts, catheters, prosthetic joints, intrauterine devices, sutures and contact lenses poses a critical problem of infection [5]. The use of intravascular catheters for patient care can give rise to central line-associated blood stream infection (CLABSI), and approximately 250,000 cases of primary blood stream infections are reported each year in the USA [6]. Thus, CLABSI results in significant morbidity and mortality and huge increases in healthcare costs. The bacteria most frequently associated with healthcare-associated infections include *Staphylococcus aureus*, *Staphylococcus epidermidis*, *Enterococcus faecalis*, *Escherichia coli*, *Klebsiella pneumoniae*, *Proteus mirabilis*, *Pseudomonas aeruginosa* and *Acinetobacter* spp. [5,7]. Among the biofilm-forming bacteria, *S. aureus* and *S. epidermidis* are predominantly isolated from cardiovascular devices [8,9]. It has been estimated that *S. aureus* and *S. epidermidis* contribute to 40–50% of prosthetic heart valve infections and 50–70% of the catheter biofilm infections [10]. In recent years, *Acinetobacter* spp. have emerged as the most important nosocomial pathogens involved in a variety of nosocomial infections, including bacteremia, urinary tract infection, soft-tissue infections and secondary meningitis [11,12,13,14]. The *Acinetobacter* spp. have the ability to colonize and form biofilms on medical devices such as implants, cardiac valves, artificial joints, catheters, etc. [11,15].

Biofilm formation is initiated when the cells attach and adhere to surfaces. The attachment of microbial cells to biomaterials can be facilitated by factors such as bacterial motility, increased shear forces, and hydrodynamic and electrostatic interactions between the microorganism and surface [16]. The adherence of bacteria to biomaterials through cell-surface and biomaterial-surface interactions is mediated by multiple factors, which include cell surface proteins, capsular polysaccharide/adhesin, protein autolysin, etc. [17,18]. For example, Staphylococcal species display cell-surface proteins, namely staphylococcal surface protein-1 and -2 (SSP-1 and SSP-2) [17], which are essential for adhesion of *S. epidermidis* to polystyrene [19]. In addition, host factors can also mediate the adherence of bacterial cells to implants, as the implant surfaces are usually covered by host plasma and other extracellular components [20]. Once attached to the surfaces, the bacterial cells will proliferate, aggregate and differentiate into biofilm structures [21]. Bacterial cells can detach from mature biofilms and spread to other organ systems, thereby contributing to persistent chronic infections [21,22].

Biofilms are complex structures with customized living environments with differing pH, nutrient availability and oxygen [23]. A worrying feature of biofilm-based infections is the increased tolerance of biofilm cells to biocides compared to planktonic bacteria [24]. The increased drug resistance could be attributed to plasmids containing genes for multidrug resistance, as biofilms form an ideal niche for plasmid exchange [25]. The mechanisms by which biofilms represent increased drug resistance also include slow or incomplete penetration of antimicrobial agents through the extracellular polymeric matrix, the formation of persister or dormant cells in a spore-like non-dividing state, slow growth rate of cells in the biofilm, thereby reducing the number of targets for antimicrobial molecules, etc. [26,27,28]. In addition to the difficulty in treating biofilm with conventional antimicrobial therapy, the treatment is further hindered by increased antibiotic resistance, as bacterial cells acquire resistance under antibiotic selective pressure [29]. For example, it has been reported that more than 70% of hospital isolates of *S. epidermidis* are methicillin resistant [30]. Thus, there is a high demand for alternative strategies to control biofilm-based infections other than antibiotic therapy. Considering the number of patients suffering from biofilm-based device-related infections, several strategies have been developed in the past few decades. This review will discuss the most successful antibiofilm approaches so far, as well as some of the more promising prospects for the control of these biofilm-based infections.

## 2. Strategies for the Control of Biofilms

There have been three major strategies considered so far to control biofilm formation or to target different stages of biofilm development. The first approach is inhibiting the initial attachment of bacteria to biofilm-forming surfaces, thereby reducing the chances of biofilm development. The second approach targets the disruption of biofilm during the maturation process [31]. The third strategy is the signal interference approach, in which the bacterial communication system or the quorum sensing (QS) system is interfered with as QS coordinates biofilm formation/maturation in pathogenic bacteria [32]. The different antibiofilm strategies and agents discussed in this review are summarized in Table 1.

### 2.1. Inhibition of Initial Attachment

The initial attachment of cells to the biofilm-forming surfaces happens within an average of the first 2 days of biofilm formation. Inhibition of initial attachment of cells to the surfaces is a potential strategy to prevent biofilm formation rather than targeting the dispersal of established biofilms. The attachment of bacteria to surfaces is mediated by several factors, including adhesion surface proteins, pili or fimbriae, and exopolysaccharides [107,108]. The surfaces that are rough, coated with surface conditioning films and more hydrophobic are prone to ease biofilm formation [109,110,111]. Thus, the initial attachment of cells can be prevented by altering the chemical or physical properties of indwelling medical devices.

#### 2.1.1. By Altering the Chemical Properties of Biomaterials

The commonly used chemical methods to modify the surface of biomedical devices in order to prevent biofilm formation include antibiotics, biocides and ion coatings [33]. Catheters coated with antibiotics such as minocycline and rifampin have been shown to decrease the incidence of biofilm-associated bloodstream infection by *S. aureus* in healthcare [34]. In addition, catheters impregnated with different antibiotics, including nitrofurazone, gentamicin, norfloxacin, etc., are suggested to have a role in preventing biofilm-associated urinary tract infections [35].

High-throughput screening of chemical libraries has led to the identification of several small chemical molecules as potential drug candidates for controlling biofilm formation and infection. These molecules do not elicit antimicrobial activity, and thus decrease the likelihood of the development of resistance due to the absence of selective pressure against biofilm formation. In *Streptococcus pyogenes* and *S. aureus*, a series of small molecules inhibited the expression of many key virulence factors that are involved in biofilm formation and infection [112,113]. The early stages of biofilm formation in *S. aureus*, *S. epidermidis* and *E. faecalis* were inhibited by several aryl rhodamines [114]. In *Vibrio cholerae*, small molecules inhibited the induction of cyclic di-GMP, which is a second messenger controlling the switch between planktonic and sessile lifestyle of bacteria [115,116]. In addition, *N*-acetylcysteine, a mucolytic agent, was reported to inhibit the production of exopolysaccharides in biofilms in *S. epidermidis* [36].

Several antimicrobial peptides are also known to interfere with biofilm formation in different bacterial pathogens. For example, peptide 1018 is considered to be a biofilm inhibitor in *P. aeruginosa*, *E. coli*, *A. baumannii*, *K. pneumoniae*, *S. aureus*, *Salmonella typhimurium*, *Burkholderia cenocepacia* [37]. In addition, lantibiotics (nisin, subtilin, epidermin and gallidermin), a class of peptide antibiotics, are reported to inhibit biofilm formation in *S. aureus*, *Lactococcus lactis* and *S. epidermidis* [38,39].

Chelators that interfere with the function of metal ions in biofilm formation are also considered to be biofilm inhibitors [117]. Metallic silver, silver salts, and silver nanoparticles have been widely used as antimicrobial agents in medical implants against bacteria such as *E. coli*, *S. aureus*, *Klebsiella* species, *P. aeruginosa*, *S. typhimurium*, and *Candida albicans* [118,119]. The silver treatment inhibits the replication of DNA, expression of ribosomal and cellular proteins, and respiration process, leading to cell death [40,41,42]. It has been reported that silver ion-coated implants inhibited *S. aureus* biofilm formation without causing silver accumulation in host tissues [120]. In addition, in the presence of nanoparticles, antibiotics such as penicillin G, amoxicillin, erythromycin, clindamycin, and vancomycin displayed increased antibacterial activity against *S. aureus* [121].

The antibacterial agent coatings on medical devices are typically effective for a short time period due to the leaching of the agent over the course of time [33]. Thus, the immobilization of antimicrobial agents on device surfaces using long, flexible polymeric chains has been an effective contribution in controlling biofilm formation in the long run. For example, the attachment of *N*-alkylpyridinium bromide, an antibacterial agent, to a polymer, poly(4-vinyl-*N*-hexylpyridine) was capable of inactivating 99% of *S. epidermidis*, *E. coli*, and *P. aeruginosa* on medical devices [43].

#### 2.1.2. By Changing the Physical Properties of Biomaterials

Biofilm formation begins with a weak reversible adhesion of bacterial cells to the surface of medical devices. If bacteria are not immediately detached from the surface of devices, they anchor permanently, using cell adhesion structures such as pili, and form biofilms [44]. Hydrophobicity and surface charge of implant materials play an important role in determining the ability of bacteria to anchor to surfaces [43]. Thus, modification of the surface charge and hydrophobicity of polymeric materials using several backbone compounds and antimicrobial agents has proven to be effective for biofilm prevention [43]. Hydrophilic polymers such as hyaluronic acid [45] and poly *N*-vinylpyrrolidone [46] on polyurethane catheters and silicone shuts, respectively, have been known to reduce the adhesion of *S. epidermidis*. In addition, various hydrogel coatings which reduce bacterial adhesion due to their hydrophilic properties have also been developed especially for ureteral stents [47]. Superhydrophobic surfaces are reported to reduce bacterial adhesion and biofilm formation due to their extremely low wettability [49,122,123]. Tang et al. observed reduced adherence of *S. aureus* on superhydrophobic titanium surfaces [124]. Also, the adhesion of *S. aureus* and *P. aeruginosa* was significantly reduced on superhydrophobic fluorinated silica coating [125]. Crick et al. demonstrated reduced adhesion of *S. aureus* and *E. coli* on AACVD (aerosol assisted chemical vapor deposition)-coated superhydrophobic surfaces compared to uncoated plain glass [123]. It has been reported that heparin interferes with bacterial adhesion and colonization [48]. The heparin coating makes the vascular catheter negatively charged, thereby preventing thrombosis and microbial colonization, eventually contributing to reduction of catheter-related infections [126,127]. Surface roughness can also influence biofilm formation, as rough, high-energy surfaces are more conducive to biofilm formation compared to smooth surfaces [128]. It is noted that the surface roughness can alter the hydrophobicity, thus in turn affecting bacterial adherence [128].

### 2.2. Biofilm Removal

Mature biofilms are highly tolerant to antimicrobials due to the altered growth rate of cells in the biofilm and the emergence of resistant subpopulations [129,130]. Also, biofilms favor the horizontal transfer of antibiotic resistance genes among cells [131]. Thus, it is of utmost importance to understand the antibiotic resistance properties of strains in biofilms when designing new drug treatments. Though conventional antibiotics have been proven to be critical in eliminating bacterial pathogens, they extensively damage the host microbiota, making the environment favorable for opportunistic pathogens. Hence, the agents that interfere with the initial biofilm development or biofilm structure have great potential in controlling biofilm-related infections.

#### 2.2.1. Matrix-Degrading Enzymes

The biofilm matrix is usually composed of exopolysaccharides (EPS), extracellular DNAs (eDNAs), and proteins [132,133,134]. The EPS and eDNAs contribute to antibiotic resistance by preventing the diffusion of antimicrobials or by inducing antibiotic resistance [135,136]. Dissociation of the biofilm matrix is an effective antibiofilm approach, as the matrix accounts for more than 90% of dry mass, and dissociation of the same will expose the sessile cells to antibiotics and host immune defence [137]. Biofilm matrix-degrading enzymes fall into three categories: polysaccharide-degrading enzymes, nucleases and proteases [50]. Dispersin B is a bacterial glycoside hydrolase produced by *Actinobacillus actinomycetemcomitans* which hydrolyzes poly-*N*-acetylglucosamine (PNAG), a major matrix exopolysaccharide of *Staphylococcus* spp. and *E. coli* [138]. In addition, the application of Dispersin B in combination with triclosan effectively reduced biofilm formation in *S. aureus*, *S. epidermidis*, and *E. coli* [51]. Endolysins, a class of peptidoglycan hydolases produced by bacteriophages are reported to digest the cell wall of bacteria thereby disrupting biofilms [52]. Deoxyribonuclease I which is capable of digesting eDNA is known to disperse biofilms in several bacteria including *Staphylococcus* strains, *A. baumannii*, *E. coli*, *Haemophilus influenzae*, *Klebsiella pneumoniae*, *Psuedomonas aeruginosa*, etc. [53,54]. The matrix proteins can be effectively cleaved by Proteinase K contributing to biofilm prevention and biofilm dispersal [55]. It was demonstrated that the treatment with dispersin B followed by Proteinase K or trypsin successfully eradicated *Staphylococcus* biofilms [55]. The *in vivo* application of matrix-degrading enzymes is limited, as the treatment can elicit inflammatory and allergic reactions in the host against these enzymes [139].

#### 2.2.2. Surfactants

Surfactants are reported to have antimicrobial and antibiofilm activities [140]. The surfactants sodium dodecyl sulfate (SDS), cetyltrimethylammonium bromide (CTAB), Tween 20 and Triton X-100 are known to promote either biofilm dispersal or detachment [56,57,58]. A biosurfactant, surfactin, which is a cyclic lipopeptide produced by *B. subtilis*, is reported to inhibit biofilm formation and induce biofilm dispersal in *S. typhimurium*, *E. coli* and *P. mirabilis* [59]. Rhamnolipids are principal glycolipids produced by many bacteria, including *P. aeruginosa*, and cause biofilm dispersal in a number bacterial strains [57,60].

#### 2.2.3. Free Fatty Acids, Amino Acids and Nitric Oxide Donors

Free fatty acids are shown to have antibiofilm activity against several pathogenic bacteria. It was reported that *P. aeruginosa* produces an organic compound *cis*-2-decenoic acid which is capable of dispersing the already established biofilms by *E. coli*, *K. pneumoniae*, *P. mirabilis*, *S. pyogenes*, *B. subtilis*, *S. aureus*, and *C. albicans* [61]. The diffusible signal factor, *cis*-11-methyl-2-decanoic acid produced by *Xanthomonas campestris* induces biofilm dispersal by controlling the production of exopolysaccharide-degrading enzyme [141]. However, it has also been reported that fatty acids play an important role in the initial stages of biofilm formation in *B. subtilis*, as the lipids form structural component of extracellular matrix of biofilms [142]. In *S. aureus*, *B. subtilis* and *P. aeruginosa*, a mixture of d-amino acids triggered the disassambly of biofilm by releasing amyloid fibers, which are the proteinaceous component of the extracellular matrix [62,63]. While many l-amino acids promote biofilm formation in *P. aeruginosa*, in the case of tryptophan, both d- and l-isoforms inhibited biofilm formation and caused biofilm dispersal [143,144]. Nitric oxide (NO) generators such as sodium nitroprusside (SNP), *S*-nitroso-l-glutathione (GSNO) and *S*-nitroso-*N*-acetylpenicillamine (SNAP) are reported to induce biofilm dispersal in *P. aeruginosa* [64]. A low dose of NO generators dispersed *P. aeruginosa* biofilms both *in vitro* and in cystic fibrosis sputum, and enhanced the effect of antibiotics on biofilm-dispersed cells [145].

### 2.3. Biofilm Inhibition by Quorum Quenching

**Figure 1:**
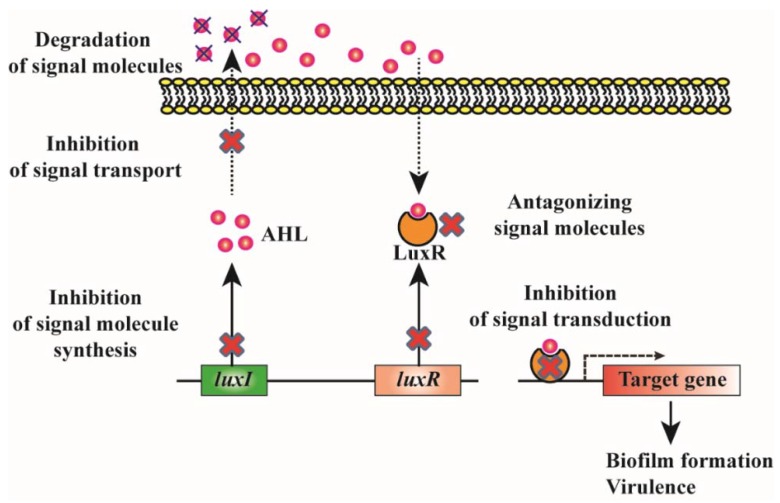
Schematic representation of various quorum-quenching strategies to control biofilm formation. *LuxI* and *luxR* genes encode AHL signal synthase and AHL receptor/activator protein respectively. AHL signal synthase is responsible for the production of AHLs, which are diffused (short chain) or pumped (long chain) out of the bacterial cell to the surrounding medium before being taken up into the nearby bacterial cells. The AHL binds to the receptor protein and the AHL-receptor complex activates the expression of quorum-sensing target genes. The quorum-quenching strategies that have been used for attenuating AHL-mediated phenotypes include the inhibition of AHL synthesis, inhibition of signal transport, degradation of signal molecules, inhibition of AHL receptor synthesis, inhibition of AHL-receptor complex formation, inhibition of the binding of AHL-receptor complex to the promoters of target genes etc.

Quorum sensing (QS) is an important cellular communication system in many Gram-negative and Gram-positive bacteria. QS mediates the regulation of various genes according to the density of signaling molecules in the surrounding environment [146]. The signaling molecules of the QS system are denoted as autoinducers [147]. Based on signaling molecules, the QS system is categorized into three; *N*-acyl homoserine lactones (AHLs)-based (Gram-negative bacteria), autoinducing peptide (AIP)-based (Gram-positive bacteria), and autoinducer-2 (AI-2)-based (both Gram-negative and Gram-positive bacteria) [148,149]. During biofilm formation, following the initial attachment, the cells secrete QS molecules, which modulate bacterial gene expression, transforming planktonic lifestyle into a sessile form [150,151,152]. Since QS plays a crucial role in biofilm formation [153], it has been suggested that QS inhibition (quorum quenching; QQ) would be an interesting strategy to prevent biofilm formation [154]. In addition, QS regulates the production of virulence factors and pathogenesis factors in most pathogens, and thus the QS system can be considered a potential target for the development of new antimicrobial agents [155,156]. The various quorum-quenching strategies that can be beneficial for controlling biofilm formation are depicted in Figure 1. The major advantage of controlling biofilm by QQ is that this strategy reduces the risk of multidrug resistance, making the strategy of great clinical interest for use in the prevention of biofilm-based infections.

#### 2.3.1. Degradation of QS Signals

AHLs can be degraded by specific enzymes such as lactonases that hydrolyze the lactone ring in the homoserine moiety and acylases that cleave off the acyl side chain, and the activity can be altered by reductases and oxidases [157]. Most of the AHL-degrading enzymes were discovered in bacterial species [158], though some are found in eukaryotes [159,160]. It has been reported that the application of QQ enzymes inhibits biofilm formation in several bacterial strains [65,66,67,68,69,70]. Quorum-quenching enzymes disrupt the biofilm architecture, which increases the antibiotic susceptibility of the cells [71]. Significant reduction of biofilm formation and increased sensitivity to antibiotics was noticed in *P. aeruginosa* after treatment with lactonase [71]. The oxidoreductases reduced the signaling molecules AHL and AI-2 to QS-inactive hydroxy-derivatives in *K. oxytoca* and *K. pneumoniae* [70].

#### 2.3.2. Inhibition of Signal Synthesis

Several reports have shown that mutations affecting AHL synthesis have an adverse effect on biofilm formation. For example, *P. aeruginosa* strain that lacked the production of 3-oxo-C12-HSL resulted in impaired biofilm formation [161]. The mutation in the gene encoding for AHL synthesis enzyme in *B. cenocepacia* K56-2, *B. cenocepacia* J2315, *Aeromonas hydrophila* and *Serratia liquefaciens* led to defective biofilm formation [162,163,164,165]. In addition, the mutants of several *Vibrio* spp., *Streptococcus* spp. and *Staphylococcus* spp. that are deficient in AI-2 synthesis were not able to produce biofilms properly. Thus, blocking signal production has been considered as a promising strategy to control biofilm formation. Analogues of AHL precursor molecule, *S*-adenosyl-methionine (SAM), such as *S*-adenosyl-homocysteine (SAH), sinefugin, 5-methylthioadenosine (MTA), and butyryl-SAM, are known to inhibit biofilm formation in *P. aeruginosa* [72]. Also, the SAM biosynthesis inhibitor cycloleucine is reported to inhibit AHL production [73]. The antibiotic azithromycin interferes with signal synthesis in *P. aeruginosa*, and thus significantly clears biofilm in mouse model of cystic fibrosis [74,75]. In addition, several inhibitors for the key enzymes (5′-methylthioadenosine/*S*-adenosylhomo-cysteine nucleosidase (MTAN) and *S*-ribosylhomocysteinase (LuxS) involved in AI-2 synthesis are shown to reduce biofilm formation [76,77]. In *B. multivorans*, nickel (Ni^2+^) and cadmium (Cd^2+^) inhibited the expression of genes responsible for AHL production thereby inhibiting cell-cell signaling and subsequently biofilm formation [79]. The inhibitory effect of Cd^2+^ in quorum sensing was also reported in *Chromobacterium violaceum* [78].

#### 2.3.3. Antagonizing the Signal Molecules

Researchers have screened for many signal analogues that antagonize QS signaling, thereby preventing biofilm formation [166,167,168]. AHL analogues in which the lactone ring was replaced by a cyclopentyl or a cyclohexanone ring adversely affected biofilm formation in *Serratia marcescens* and *P. aeruginosa* [169,170]. Many natural compounds are also reported to antagonize AHL-based QS signaling, and those include bergamottin and dihydroxybergamottin from grapefruit juice, cyclic sulfur compounds from garlic, patulin, and penicillic acid from a variety of fungi, etc. [80,81,82]. Treatment with patulin, ajoene and garlic extracts resulted in increased antibiotic susceptibility of *P. aeruginosa* biofilms and increased clearance of *P. aeruginosa* in *in vivo* pulmonary infection model [83,84,85]. In addition, some phenolic compounds including baicalin hydrate and epigallocatechin blocked AHL QS and affected biofilm formation of *B. cenocepacia*, *B. multivorans* and *P. aeruginosa* [86,87,88]. It was noted that the antibiotic susceptibility of *B. cenocepacia* and *P. aeruginosa* increased after treatment with baicalin hydrate in different *in vitro* biofilm models [86,87,88]. Thus, the concept of combining QS inhibitor (QSI) and antibiotics would be a better strategy to control biofilm formation by pathogenic bacteria. In addition, it has been noticed that biofilm formation can be effectively controlled by combining QSIs and QQ enzymes. Recently, Fong et al. reported the synergistic effect of a QS inhibitor, G1, which competes with AHL to bind to the response regulator and QQ enzyme, AHL lactonase, to effectively control biofilm formation and virulence by *P. aeruginosa* [171].

Several compounds that antagonize AI-2 signaling have also been reported to exhibit antibiofilm activity. The AI-2 analogues ursolic acid, isobutyl-4,5-dihydroxy-2,3-pentanedione (isobutyl-DPD) and phenyl-DPD inhibited biofilm formation and removed preformed biofilms in *E. coli* and *P. aeruginosa* [89,90]. Although other compounds, including pyrogallol and its derivatives, some nucleoside analogues, boronic acids, and sulfones have been identified to antagonize AI-2 signaling, only a few have been investigated for their antibiofilm activity [172,173].

Several AIP analogues, such as truncated forms of AIP, and probiotic bacteria-producing natural cyclic dipeptides, such as cyclo(l-Phe-l-Pro) and cyclo(l-Tyr-l-Pro), have been developed to antagonize QS signaling in Gram-positive bacteria. However, the experimental evidence on the anti-biofilm activities of these compounds is highly limited [91,92,93]. The most investigated QS inhibiting peptide is the RNAIII inhibiting peptide (RIP), which is produced by coagulase-negative *Staphylococci*. RIP interferes with the QS response by inhibiting the production of RNAIII, a key component of QS response in *S. aureus* [94]. RIP and several RIP homologues have been reported to have anti-QS and anti-biofilm activity against *Staphylococcus* spp. A RIP analogue, FS3, prevented *S. aureus* biofilm formation in a rat vascular graft model [95]. In addition, a non-peptide RIP analogue, hamamelitannin, blocked QS in *Staphylococcus* spp., and potentially inhibited biofilm formation in *in vitro* and *in vivo* rat model of graft infection [96]. Several natural compounds, including phytol, anthocyanidins, extracts from *Ricinus communis*, freshwater bryozoan *Hyalinella punctata* and selected sponges, and ricinine derivatives, are also known to exhibit anti-biofilm or anti-microbial and anti-quorum sensing activities in *P. aeruginosa* [174,175,176,177,178]. However, the exact mechanisms by which these compounds display anti-quorum sensing activities are not known.

#### 2.3.4. Inhibition of Signal Transduction by Interfering with Response Regulator Activity

**Figure 2:**
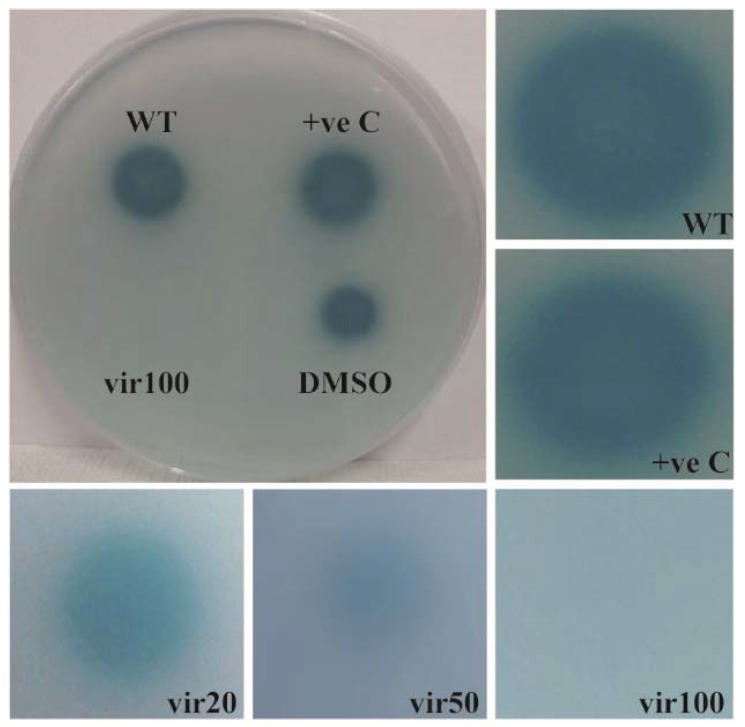
Effect of virstatin on the production of AHL. Bioassay was carried out to check the effect of virstatin on the production of AHLs in *A. nosocomialis*. For this, the strain was cultivated overnight in Luria Bertani (LB) medium at 30 °C, and the cells were washed with LB and diluted to an OD_600_ of 1. The cells were treated with different concentrations of virstatin (20, 50, 100 mM), which was dissolved in dimethyl sulfoxide (DMSO), and 5 µL of the samples were spotted onto chromoplate overlaid with *A. tumefaciens* NT1 (pDCI41E33) [184,185]. Synthetic *N*-(3-hydroxy-dodecanoyl)-l-homoserine lactone (OH-dDHL) was spotted as a positive control. The plates were incubated at 30 °C for 22 h, followed by the detection of the color zone surrounding the bacteria. A representative chromoplate image with 100 mM virstatin and images of color zones from different concentrations of virstatin are shown. WT, *A. nosocomialis* wild type; +ve C, OH-dDHL; vir20, vir50 and vir100; wild-type cells treated with 20, 50 and 100 mM virstatin respectively.

The QS system can also be hindered at the level of signal transduction cascade. The natural compounds, halogenated furanone or fimbrolide and cinnamaldehyde which are isolated from red algae *Delisea pulchra* and cinnamon bark, respectively, interfere with signal transduction and affect biofilm formation, thereby increasing antibiotic susceptibility in several pathogenic bacteria [97,98,99,100]. Both compounds block AI-2 and AHL-type QS systems, and thereby affect biofilm formation in *V. harveyi* [101,102]. The halogenated furanone and cinnamaldehyde inhibits AI-2 QS and AHL QS by decreasing the DNA-binding ability of the response regulator LuxR, which is important for the signal transduction cascade, or by displacing AHL from its receptor, respectively [101,102,179,180]. In addition, the natural furanone inactivates LuxS and accelerates LuxR turnover, thereby blocking AI-2 and AHL QS signaling system, respectively [181,182]. Cinnamaldehyde is widely used as a flavoring agent in food and beverages, while the application of furanones is limited because of their toxicity [100,183]. We reported previously that virstatin, a small organic molecule, prevents biofilm formation by interfering with the QS system in *A. nosocomialis* [103]. It was noticed that virstatin inhibits the expression of the response regulator, AnoR, which is a positive regulator of the AHL synthase gene, *anoI* in *A. nosocomialis* [103]. The repression of AnoR leads to decreased synthesis of AHL (Figure 2), adversely affecting the signal transduction cascade. Virstatin or its derivatives can be considered potential agents to inhibit the QS system and to control biofilm-based infections, and further studies in this direction could lead to the development of better antibacterial therapeutics.

#### 2.3.5. Inhibition of Signal Transport

The signaling molecules need to be exported and released into the extracellular space to be sensed by other bacteria for effective cell-to-cell communication. The role of multidrug-resistant (MDR) efflux pumps in signal traffic was first reported in *P. aeruginosa*, in which AHLs with long side chains are actively transported across the cell membrane through the MexAB-OprM efflux pump [186]. In *P. aeruginosa*, the expression of the autoinducer-producing gene and the genes encoding the virulence factors is limited by the intracellular concentration of the autoinducer [187]. The involvement of the MDR efflux pump in the QS system has also been reported in *E. coli*, in which the overexpression of the QS regulator SdiA led to the increased expression of the AcrAB efflux pump [188]. In *Bacteroides fragilis*, an opportunistic pathogen of the gastrointestinal tract, the BmeB efflux pump controls the intracellular AHL concentration by effluxing AHL outside of cells [189]. In addition, the expression of the MDR efflux pump, BpeAB-OprB, was reported to be essential for the export of six AHL inducers to the extracellular environment in *B. pseudomallei* [190,191]. Thus, the inhibition of the efflux pump would be a promising strategy to alter QS signaling cascade, thereby preventing biofilm formation and virulence.

Several studies have provided evidence to show the link between the physiological function of efflux pump and biofilm formation. In *E. coli* and *Klebsiella* strains, the inhibition of the efflux pump activity using efflux pump inhibitors (EPIs) reduced biofilm formation [192]. The genetic inactivation or the chemical inhibition of efflux pump activity resulted in impaired biofilm formation in *S. enterica* serovar *typhimurium* [193]. The effect of efflux pump inhibitors to prevent biofilm formation was also demonstrated in *P. aeruginosa* and *S. aureus* [105], in which copper nanoparticles work well as EPI and anti-biofilm agents [104]. In addition, in *P. aeruginosa*, the MDR efflux pump, MexAM-OPrM was disrupted by silver nanoparticles [194]. Recently, it was observed that the well-characterized EPI, Phe-Arg-β-naphthylamide (PAβN) alter the expression of QS molecules and QS-dependent virulence phenotypes in *P. aeruginosa* PAO1, as well as in clinical isolates [106]. The application of EPIs not only helps to reduce the biofilm-forming capacity of bacteria, but also to revive the bactericidal effect of conventional antibiotics [195].

It has been reported previously that AHLs with long side chains are exported out through the MexAB-OprM pump in *P. aeruginosa* [186], and the expression of the pump is modulated by the intracellular concentration of autoinducer molecules [196]. In addition, we have identified previously that *A. nosocomialis* produces AHL with long side chain, *N*-(3-hydroxy-dodecanoyl)-l-homoserine lactone (3OH-C12-AHL) as signaling molecules [103], and these AHLs might be actively transported through the efflux pumps. Thus, it can be postulated that the QS system controls the activity of the efflux system, contributing to the effective transport of AHLs across the cell membrane, in turn contributing to virulence and biofilm formation. Further studies in this direction would unravel in depth the role of the QS system in controlling the activity of these efflux pumps. The MDR efflux pumps and the regulators modulating them would be potential targets for the development of better therapeutics for biofilm-based infections.

## 3. Conclusions

In this review, we discuss the current strategies and future perspectives for developing improved therapeutics for controlling biofilm-based infections. The various approaches for modulating biofilm formation on medical devices are addressed in detail, with special emphasis on quorum-quenching strategies. Significant advances have been made in understanding the role of quorum sensing in biofilm formation in the past few years. In addition, several studies have shown that multidrug efflux pumps play a potential role in controlling biofilm formation. However, the fundamental mechanisms by which the QS systems exert the regulatory functions on biofilm formation are poorly understood. In this review, we postulate that QS systems regulate the activity of multidrug efflux pumps in transporting QS molecules across the cell membrane, thereby affecting biofilm formation. We propose that the transcriptional factors modulating the QS system and/or efflux pumps would be potential targets for developing QSIs. It is of utmost importance to improve our understanding of the molecular mechanism by which QS systems regulate biofilm formation and multidrug efflux pumps, as it will eventually be of help in developing better therapeutics for the treatment of problematic biofilm-related infections. Furthermore, detailed research is needed to understand the effect of these QSIs on different stages of biofilm formation and to validate their applicability on humans. Since QSIs do not induce any antibiotic resistance, they can be of great potential in the future for the treatment of biofilm-based infections in healthcare settings.

## Figures and Tables

**Table 1:** Various strategies for the control of biofilms.

Strategy	Methods/Agents	Examples	References
Inhibition of initial biofilm attachment	(i) Altering chemical properties of biomaterials	(i) Antibiotics, biocides, iron coatings	(i) [33,34,35,36,37,38,39,40,41,42,43]
	(ii) Changing physical properties of biomaterials	(ii) Use of hydrophilic polymers, superhydrophobic coatings, hydrogel coatings, heparin coatings	(ii) [44,45,46,47,48,49]
Removal of biofilms	(i) Matrix degrading enzymes	(i) Polysaccharide-degrading enzymes (Dispersin B, Endolysins); Nucleases (Deoxyribonuclease I) and Proteases (Proteinase K, trypsin)	(i) [50,51,52,53,54,55]
	(ii) Surfactants	(ii) Sodium dodecyl sulfate (SDS), cetyltrimethylammonium bromide (CTAB), Tween 20 and Triton X-100, surfactin, rhamnolipids	(ii) [56,57,58,59,60]
	(iii) Free fatty acids, amino acids and nitric oxide donors	(iii) *Cis*-2-decenoic acid, d-amino acids, nitric oxide generators such as sodium nitroprusside (SNP), *S*-nitroso-l-glutathione (GSNO) and *S*-nitroso-*N*-acetylpenicillamine (SNAP)	(iii) [61,62,63,64]
Biofilm inhibition by quorum quenching	(i) Degradation of QS signals	(i) Lactonases, acylases and oxidoreductases	(i) [65,66,67,68,69,70,71]
	(ii) Inhibition of signal synthesis	(ii) Use of analogues of AHL precursor *S*-adenosyl-methionine (SAM), *S*-adenosyl-homocysteine (SAH), sinefugin, 5-methylthioadenosine (MTA), butyryl-SAM; SAM biosynthesis inhibitor cycloleucine, AHL synthesis inhibitors such as nickel and cadmium	(ii) [72,73,74,75,76,77,78,79]
	(iii) Antagonizing signal molecules	(iii) AHL analogues (bergamottin, dihydroxybergamottin, cyclic sulfur compounds, phenolic compounds including baicalin hydrate and epigallocatechin); AI-2 analogues (ursolic acid, isobutyl-4,5-dihydroxy-2,3-pentanedione (isobutyl-DPD) and phenyl-DPD); AIP analogues (cyclic peptides such as cyclo (l-Phe-l-Pro) and cyclo(l-Tyr-l-Pro), RNAIII inhibiting peptide (RIP) and its homologues)	(iii) [80,81,82,83,84,85,86,87,88,89,90,91,92,93,94,95,96]
	(iv) Inhibition of signal transduction	(iv) Use of halogenated furanone or fimbrolide, cinnamaldehyde, virstatin	(iv) [97,98,99,100,101,102,103]
	(v) Inhibition of signal transport	(v) Use of copper or silver nanoparticles, Phe-Arg-β-naphthylamide (PAβN)	(v) [104,105,106]
